# Fatal anti-aquaporin-4 seropositive neuromyelitis optica spectrum disorder in tuberculosis

**DOI:** 10.1186/1471-2334-14-470

**Published:** 2014-08-28

**Authors:** Siddharth Sridhar, Chan Jasper Fuk-Woo, Kwok-Yung Yuen

**Affiliations:** Department of Microbiology, The University of Hong Kong, Queen Mary Hospital, 102 Pokfulam Road, Pokfulam, Hong Kong Special Administrative Region, China; State Key Laboratory of Emerging Infectious Diseases, The University of Hong Kong, 102 Pokfulam Road, Pokfulam, Hong Kong Special Administrative Region, China; Research Centre of Infection and Immunology, The University of Hong Kong, 102 Pokfulam Road, Pokfulam, Hong Kong Special Administrative Region, China

**Keywords:** Tuberculosis, Neuromyelitis optica, Anti-aquaporin-4 antibody

## Abstract

**Background:**

Neuromyelitis optica (NMO) is an autoimmune inflammatory condition of the central nervous system that is characterized by circulating anti-aquaporin-4 antibodies, transverse myelitis and optic neuritis. NMO spectrum disorders are rarely reported in patients with active pulmonary tuberculosis (TB). We report a fatal case of anti-aquaporin-4 antibody positive NMO spectrum disorder in a patient who was receiving treatment for pulmonary tuberculosis.

**Case presentation:**

A previously healthy 42-year-old Chinese man was diagnosed with smear positive pulmonary tuberculosis. After one month of anti-tuberculosis treatment, he presented with acute generalized weakness and rapid neurological deterioration. Spinal imaging and anti-aquaporin-4 antibody positivity established a diagnosis of neuromyelitis optica spectrum disorder.

**Conclusion:**

This is the first reported case of anti-aquaporin-4 antibody-positive NMO spectrum disorder in a patient with active tuberculosis. It shows the usefulness of testing for anti-aquaporin-4 antibodies while evaluating neurological deterioration in patients with tuberculosis. The literature on the rare association between NMO spectrum disorders and TB is reviewed.

**Electronic supplementary material:**

The online version of this article (doi:10.1186/1471-2334-14-470) contains supplementary material, which is available to authorized users.

## Background

Neuromyelitis optica is an autoimmune inflammatory disorder of the central nervous system affecting the optic nerves and the spinal cord, manifesting as recurrent episodes of longitudinally extensive transverse myelitis (LETM) and/or optic neuritis. Previously classified as a subtype of multiple sclerosis, NMO is now considered to be a distinct clinical, pathological, and immunological entity [1]. A key diagnostic test for NMO spectrum disorders is the presence of anti-aquaporin-4 (anti-Aqp-4) IgG antibodies in patient serum [2]. Aquaporin-4 is a membrane water channel that is most abundant at astrocytic end-feet. Anti-Aqp-4 IgG has been found to be a highly specific marker for the NMO spectrum disorders that include classic NMO, isolated optic neuritis, isolated LETM, and isolated brain stem encephalitis [3–6]. This test now forms part of the diagnostic criteria for neuromyelitis optica [2].

A small number of case reports describe patients developing NMO like syndromes shortly after being diagnosed with pulmonary tuberculosis, hinting at a rare association between the two entities [7–9]. Despite the shortcomings of case–control studies in elucidating rare associations between exposures and disease, two groups have conducted case–control studies comparing the prevalence of TB between NMO patients and control patients with other neurological diagnoses. While one study found a significant association between NMO and TB in a high TB – low NMO prevalence setting in South Africa, another study in an intermediate TB – high NMO prevalence setting in southern China failed to demonstrate a significant association [10, 11]. However, a clinical trial conducted in another Chinese NMO patient group demonstrated a marked beneficial effect of anti-TB treatment on the course of patients with steroid refractory NMO even in the absence of overt tuberculosis suggesting a possible association of latent TB with NMO that would have been missed in the case–control studies [12]. Therefore, the evidence to date is in favor of an association with TB in a subset of NMO patients, who may benefit from anti-TB treatment. None of the patients with TB associated NMO-like disorder described previously have been shown to have anti-Aqp-4 antibodies.

We report a rapidly progressive and fatal case of anti-Aqp-4 antibody-positive neuromyelitis optica (NMO) like disorder in a patient receiving treatment for pulmonary TB. To the best of our knowledge, our case is the first report of anti-Aqp-4 antibody-positive NMO like disorder in a patient with active pulmonary TB. The rapidity of our patient’s deterioration is also unprecedented in the published literature on TB-associated NMO.

From our case report and other cases in the literature, NMO appears to develop shortly after the diagnosis of pulmonary TB even when the patient is started on appropriate anti-TB medications. We show that anti-Aqp-4 antibodies are present in TB associated NMO; these antibodies are known to play a pathogenic role in NMO [2]. The mechanism by which TB infection triggers the formation of these antibodies is uncertain. *Mycobacterium tuberculosis* surface antigens may trigger the formation of cross-reactive antibodies against aquaporin-4 channel proteins. However, immunity against active TB infection is generally geared towards a cell-mediated immune response rather than a humoral response, therefore the contribution of this mechanism is uncertain. Further research is required to elucidate the biological basis for this association.

In summary, this case highlights NMO like disorder as a fatal immunological complication of TB and illustrates the diagnostic role of anti-Aqp-4-antibody in patients with active TB and concomitant neurological deterioration.

## Case presentation

**Figure 1:**
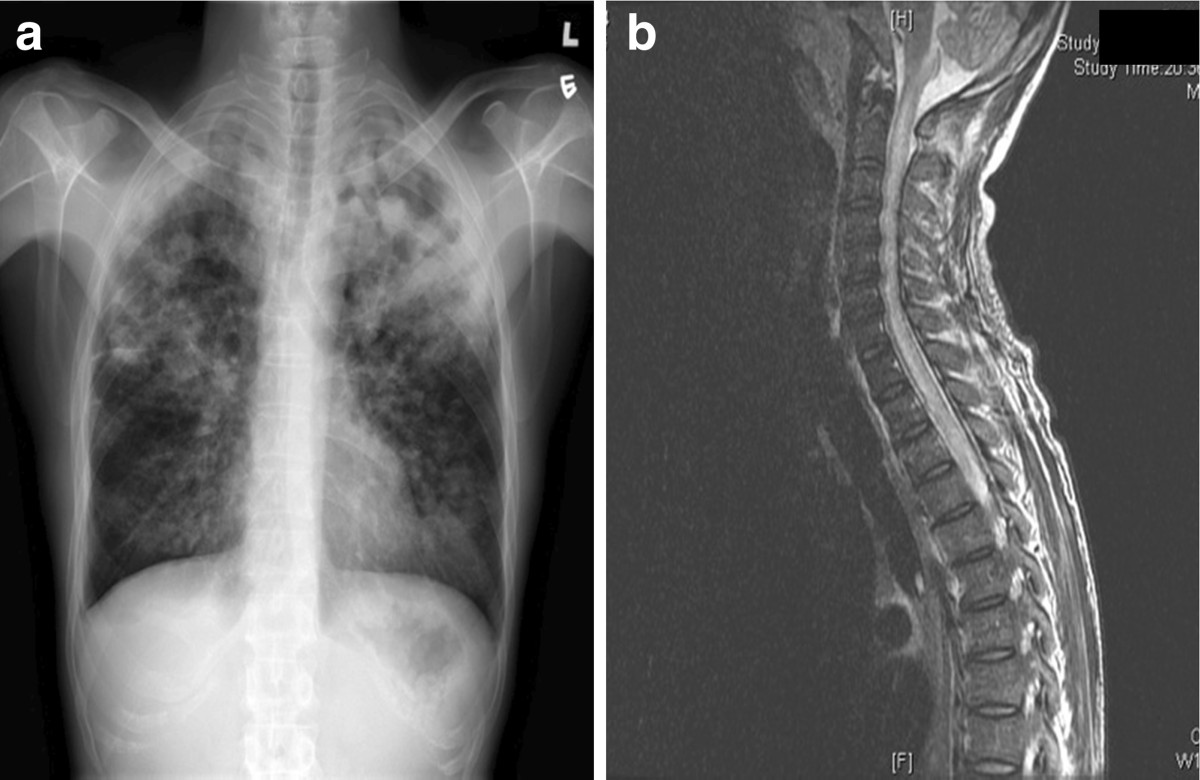
**Radiological imaging of lung and spinal cord. a**: Chest X-ray (PA Erect film) on initial presentation showing bilateral lung field patchy consolidation with left upper zone predominance. **b**: Sagittal section of MRI spine showing diffuse T2-weighted hyperintensity involving C1-T4 levels of the spinal cord suggestive of longitudinally extensive transverse myelitis (LETM).

A previously healthy 42-year-old man presented with cough and fever for three weeks. Chest radiograph on admission revealed bilateral consolidations with predominant upper zone involvement (Figure 1a). Blood tests showed normal total white cell count (5.99×10^9^/l), but marked lymphopenia (0.20×10^9^/l). Ziehl-Neelsen-stained smear of the sputum revealed >1 acid-fast bacillus (AFB) per oil-immersion field, suggestive of pulmonary tuberculosis (TB). The patient was started on oral isoniazid 300 mg daily, rifampicin 450 mg daily, ethambutol 700 mg daily, pyrazinamide 1250 mg daily, and pyridoxine 10 mg daily. His condition improved with good drug compliance under directly observed treatment in an outpatient setting. Sputum cultures on Lowenstein-Jensen and Stonebrink media were positive for *Mycobacterium tuberculosis*. The patient was readmitted after four weeks of treatment with generalized weakness. Physical examination showed weakness of MRC grade two over all four limbs. He was hyporeflexic and plantar responses were equivocal. He had a palpable bladder and lax anal tone. Cranial nerve examination was unremarkable. Blood tests showed leukocytosis with neutrophilia and lymphopenia (total white cell, 13.11×10^9^/l; neutrophils, 11.17×10^9^/l; lymphocytes, 0.49×10^9^/l). Chest radiograph showed worsened bilateral apical lung infiltrates. A magnetic resonance imaging (MRI) scan of the spine was performed urgently and revealed diffuse intramedullary T2-weighted hyperintensity of the spine from C1 to T4 levels and from T4 to the conus terminalis, suggestive of longitudinally extensive transverse myelitis (LETM) (Figure 1b). Diffuse consolidation of bilateral lung apices and a cavitating lesion with air-fluid level at the left lung apex were noted. Ziehl-Neelsen-stained smear of the endotracheal aspirate showed >1 AFB/oil immersion field, confirming treatment failure despite four weeks of first-line anti-tuberculous treatment.

The patient’s condition deteriorated rapidly with high fever, hypotension, and coma. He was intubated and vasopressor support was commenced. A plain computed tomography scan of the brain was unremarkable. Lumbar puncture showed clear cerebrospinal fluid (CSF) with an elevated opening pressure of 20 cmH_2_O. Total cell count of the CSF was elevated to 62×10^6^/l with neutrophil predominance. CSF and peripheral blood glucose levels were 1.1 mmol/l and 4.9 mmol/l respectively, and CSF protein was elevated to >6 mmol/l. Gram-stained and Ziehl-Neelsen-stained smears of the CSF showed no organism. Culture of the CSF yielded no bacterial, mycobacterial, and fungal growth. Molecular tests including TB-PCR, HSV-PCR, and enterovirus RT-PCR were negative.

A diagnosis of disseminated TB with central nervous system involvement was made. As the patient’s condition had deteriorated despite four weeks of first line anti-tuberculous drugs, multidrug-resistant TB was suspected. The drug regimen was changed to intravenous rifampicin 450 mg q24 h, ethambutol 1200 mg q24 h, levofloxacin 500 mg q24 h, linezolid 600 mg q24 h, and amikacin 750 mg q24 h. The patient was also given a combination of intravenous meropenem 1000 mg q8 h and intravenous amoxicillin-clavulanate 1200 mg q12 h for broad-spectrum antibacterial and anti-tuberculous coverage [13, 14]. Intravenous dexamethasone was also started at 4 mg q8 h [15]. Genotypic testing for drug resistance using a line probe assay (GenoType MTBDR*plus*, Hain Lifescience) on the *M. tuberculosis* isolate from the patient’s first admission was negative for the common isoniazid and rifampicin resistance mutations (*katG*, *inhA* and *rpoB* respectively). An underlying immunological defect was considered in view of the clinical deterioration despite in-vitro drug susceptibility. The combined HIV-antigen/antibody test and anti-interferon-gamma autoantibody were negative [16, 17].

Due to the radiological finding of LETM, the neuromyelitis optica (NMO) spectrum disorders were a diagnostic consideration. Serum anti-aquaporin-4 (anti-Aqp-4) antibodies were positive by an immunofluorescence assay (Euroimmun). The patient was diagnosed with NMO spectrum disorder complicating active pulmonary TB without evidence of mycobacterial central nervous system infection. Despite treatment with corticosteroid, the patient’s condition further deteriorated with severe hyperthermia, high ventilatory requirement, loss of brainstem reflexes, and labile hemodynamic status. He died after thirteen days of admission.

## Conclusion

In summary, our case highlights NMO like disorders as an important diagnostic consideration in patients with active TB who present with acute neurological disorders. Serum anti-Aqp-4 antibodies should be part of the diagnostic workup of patients with active TB who present with transverse myelitis and/or optic neuritis. Further multicenter trials are required to clarify the etiological role of TB in triggering NMO like disorders.

### Consent

Written informed consent was obtained from the patient’s next of kin (brother) for publication of this Case report and any accompanying images. A copy of the written consent is available for review by the Editor of this journal.

## Authors’ original submitted files for images

Below are the links to the authors’ original submitted files for images. Authors’ original file for figure 1
