# Supplementary figures and images for: Fatal anti-aquaporin-4 seropositive neuromyelitis optica spectrum disorder in tuberculosis
Source: BMC Infect Dis. 2014 Aug 28;14:470. doi: 10.1186/1471-2334-14-470 (PMC4158048; doi:10.1186/1471-2334-14-470)

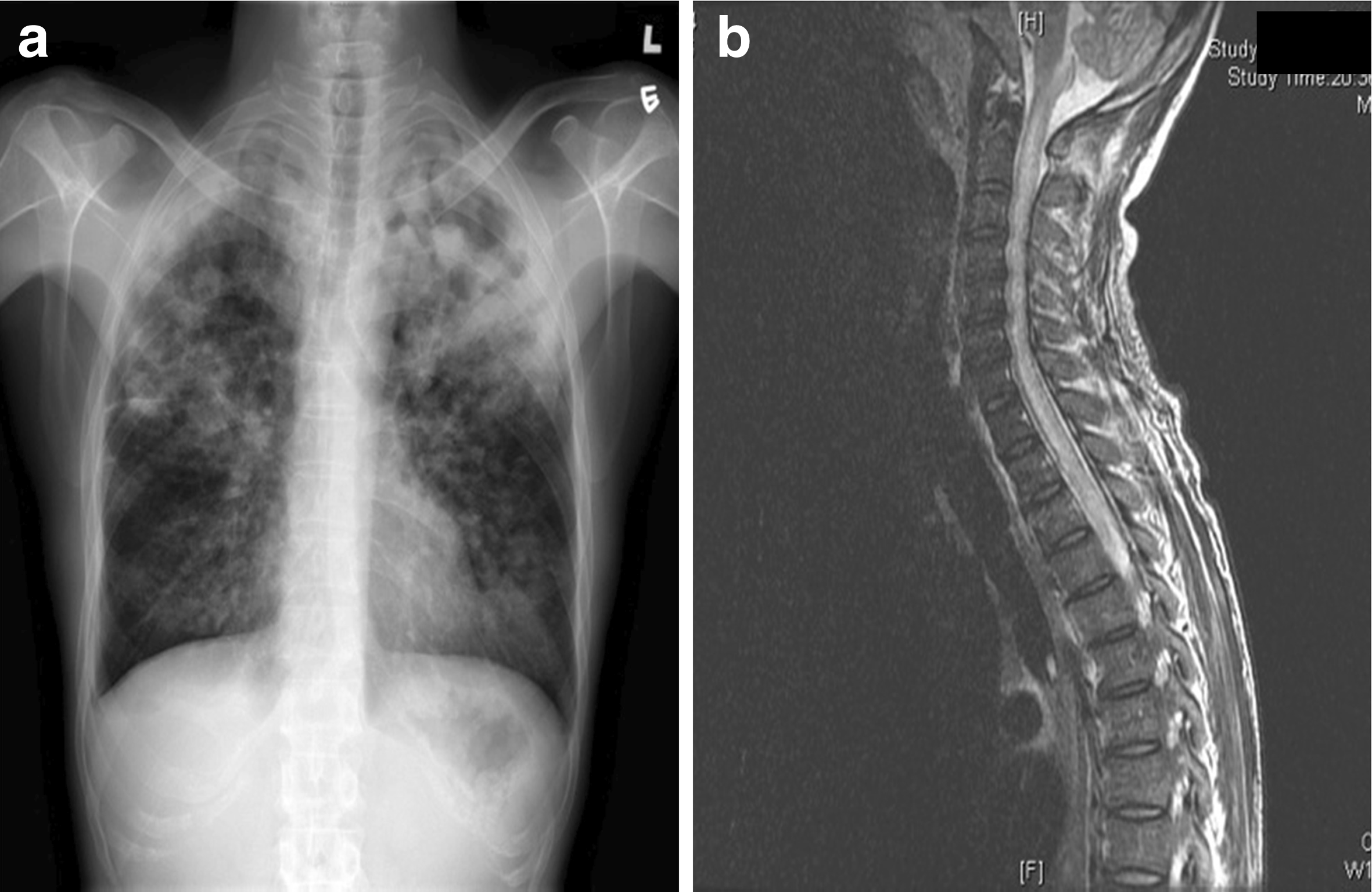

Supplement: Supplementary file 1 — Authors’ original file for figure 1 [file 12879_2014_3778_MOESM1_ESM.tif]
